# Supplementary material for: Curated single cell multimodal landmark datasets for R/Bioconductor
Source: PLoS Comput Biol. 2023 Aug 25;19(8):e1011324. doi: 10.1371/journal.pcbi.1011324 (PMC10497156; doi:10.1371/journal.pcbi.1011324)
Supplement: Attachment — Submitted filename: SCMM PLOS Comp Bio rebuttal letter.docx [file pcbi.1011324.s001.docx]

Dear Dr. Noble,

We thank you and the two referees for the feedback on our manuscript and for the opportunity to respond to the feedback. We note that all reviewers were overall positive and requested similar revisions, in particular, additional labeling of cell type and quality control information. In summary:

- Our initial submission included standardized single cell multimodal datasets from different experimental techniques; however, it was not clear whether cell type labels were included for each dataset. This was raised by both reviewers. We now include cell type information everywhere it is possible and have added Table 9 to make this information immediately available to the reader.
- Similarly, some datasets are pre-filtered by the study authors to retain only high-quality cells, while others include all unfiltered cells. We have clarified in the manuscript which of these situations applies to each dataset, and have added per-cell annotations within each dataset to indicate whether a cell is pre-filtered, or adding quality control information to allow filtering by the user.

We have highlighted in red all the changes made to the manuscript compared to the previously submitted version, and detail these changes below.

**Comments to the Authors:**

Review 1:

### The authors collected multimodal single-cell datasets from multiple human tissues and built a resource named SingleCellMultiModal. The landmark datasets and the Bioconductor package presented here will be useful for new computational method development. However, I have two concerns about the manuscript.

### *Reviewer #1: Collecting the right datasets is important for computational method development. One challenging point is that most datasets do not have cell type label information. Thus, it is difficult to evaluate the newly developed methods. There are some datasets that have great quality and have been analyzed in literature by some great methods or have been manually annotated. Collection of those datasets will be extremely useful for method development. The impact of the software/manuscript will be significantly increased if datasets with annotated cell labels will be included*.

Response:

We thank the reviewer for raising this important point and we agree that datasets with manually annotated cell type information are extremely useful for method development. In fact, while the previous version of our manuscript glossed over this information, the majority of the datasets included in *SingleCellMultiModal* do include cell type annotation.

To standardize and facilitate the retrieval of cell type information across datasets with the package, we have added a function “ontomap” and plain-text file `inst/extdata/ontomap.tsv` that provide a table of available cell type annotations, standardized where possible to the Cell Ontology (first choice) or NCI Thesaurus (when not in Cell Ontology).

The SCoPE2, seqFISH, scMultiome, and scNMT datasets now include ontology identifiers based on labels provided by the authors of the original publication. The new ontomap function is now available, starting from devel version 1.13.4 and release version 1.12.2.

While the CITEseq and ECCITEseq datasets do not formally have cell type labels, the ADT data for cell surface markers typically used in immunology allow for manual gating of cell types (see e.g., Supplementary Figure 3 of Stoeckius et al.). We used this additional information to define cell type labels for the CITEseq dataset. This additional information can be used for method development/benchmarking, by e.g., looking for maximal correspondence between scRNA-seq derived cell type labels and ADT surface markers, which can be considered as gold standard. We added two new functions (getCellGroups and addCTLabels) to help external users perform their own manual gating. We report the steps we performed to identify the cell types in the Methods as follows:

”Because this dataset provides a mixture of human and mouse cells, we applied k-means clustering combined with hierarchical clustering on the log transformed scRNA counts to identify then classify human cells, approximately reconstructing the population of cells that the landmark paper reported through manual gating on the ADT assay. Despite a few unidentified cells, most of the subgroups of cells are identified, i.e., Natural Killers, Precursors, CD4 T-cells, CD8 T-cells, B-cells, Monocytes CD14+ and Monocytes CD16+. The code for cell filtering and cell manual gating has been released inside the script folder of the SingleCellMultiModal package. Additionally, two functions (getCellGroups and addCTLabels) for the manual gating are now exported by the package to help users identify their own cell populations.”

Unfortunately, the authors of the G&Tseq dataset do not provide original cell type annotations and it is unclear how to define a gold-standard annotation for this dataset, but we argue that this dataset can still be a useful multimodal resource for methods not requiring cell type annotations.

We added these considerations at the end of the paragraph “SingleCellMultiModal data package” in the Methods section and we summarized cell-type annotations and cell quality control information in the new Table 9. Specifically, we added the following Methods text:

“All datasets provide a cell-type annotation either provided by the authors of the original publications or based on external knowledge, except for ECCITEseq and G&T-seq for which none was available (Table 9). In particular, the SCoPE2, seqFISH, and scNMT datasets include the cell type labels provided by the authors of the original publication, while scMultiome includes a cell type label inferred by manually inspecting cluster markers. While the CITEseq dataset does not formally have cell type labels, the ADT data for cell surface markers typically used in immunology allow for manual gating of cell types (see e.g., Supplementary Figure 3 of (3)). We used this additional information to define cell type labels that are independent of scRNA-seq data. We note that this dataset has been previously used for method benchmarking by looking for maximal correspondence between scRNA-seq derived cell type labels and ADT surface markers, which can be considered as gold standard (see e.g. (61)). A dedicated function (getCellGroups) has been added to the SingleCellMultiModal package to help the user to add their own cell annotations based on manual gating. An overview of the cell type annotation for all the dataset can be easily retrieved through the ontomap function, which also standardizes cell names from the Cell Ontology(62) (first preference) or NCI Thesaurus(63) to facilitate cross-dataset comparisons.”

### 2. Because of technical reason, there are always have some bad quality cells. Including too much bad quality cells will affect the performance of the computational methods. It would be helpful for users if a reasonable quality score is included.

We thank the reviewer and agree that including quality control information to the datasets is important.

In some cases, the processed data provided in the original publication was already filtered to exclude low-quality cells. When this is the case, we state it clearly in the documentation.

When no pre-filtering was done, we now provide an option to retrieve either the filtered dataset, which includes only cells that pass a quality control step, or the full dataset along with an indication, for each cell, of whether it passes the QC filters, defined following state-of-the-art best practices and specified in the documentation of each dataset/function.

In detail:

- For the SCoPE2 data sets, low-quality cells for the single-cell proteomics and scRNA-Seq data were already filtered out by the authors that generated the dataset. More specifically, cells with coefficients of variation that were similar to empty samples were removed from the proteomics data, and cells with less than 10^4^ UMI barcodes were removed from the transcriptomics data.
- For the G&Tseq dataset the original authors already computed cell quality control and released the already filtered dataset.
- For the scNMT dataset, since version 2.0.0, quality control filters computed on each modality are available in the colData of the object.
- For the CITEseq and ECCITEseq, cell filtering is now available through the colData of the objects. Quality controls have been performed following Section 12.3 of the Advanced Single-Cell Analysis with Bioconductor book ([https://bioconductor.org/books/release/OSCA/](https://bioconductor.org/books/release/OSCA/P)).
- For seqFISH cell quality preprocessing has been performed by LeCao et al. (2021) [https://doi.org/10.1186/s13059-021-02433-9], and we released the dataset as they provided it.
- For the scMultiome dataset, we provide cell quality controls in the colData of the released object.

We summarize the availability of this quality control information in the new Table 9 and added the following text to the Methods:

“Furthermore, each dataset includes information on the the quality of the cells: for some datasets, the package provides a pre-filtered object, in which low-quality cells were already excluded by the original authors; for others, we provide an option to retrieve either the filtered dataset, which include only cells that pass a quality control step, or the full dataset along with an indication, for each cell, of whether it passes the QC filters, defined following state-of-the-art best practices (Table 9).”

| EXPERIMENTAL ASSAY | DATATYPE NAME | CELL QC | VERSION QC | CELL ANNOTATION | CELL ANNOTATION COLUMN |
| --- | --- | --- | --- | --- | --- |
| G&T-seq | mouse_embryo_8_cell | Filtered as in [(7)](https://paperpile.com/c/FZKGcV/opjY) | 1.0.0 | absent | NA |
| CITE-Seq | cord_blood | Based on [(64,65)](https://paperpile.com/c/FZKGcV/zzXV+nrsc) | 1.0.0 | Manual gating on ADT markers | celltype |
| ECCITE-Seq | peripheral_blood | Based on [(64,65)](https://paperpile.com/c/FZKGcV/nrsc+zzXV) | 1.0.0 | absent | NA |
| scNMT-seq | mouse_gastrulation | Based on [(5)](https://paperpile.com/c/FZKGcV/DXhv) | 2.0.0 | Provided by | lineage |
| 10X Multiome | pbmc_10x | Based on | 1.0.0 | Manual curation based on markers | celltype |
| seqFISH | mouse_visual_cortex | unavailable | 1.0.0 | Provided by [(20)](https://paperpile.com/c/FZKGcV/0y75) | class |
|  |  | Filtered as in [(20)](https://paperpile.com/c/FZKGcV/0y75) | 2.0.0 |  |  |
| SCoPE2 | macrophage_differentiation | Filtered as in [(8)](https://paperpile.com/c/FZKGcV/aAyp) | 1.0.0 | Provided by [(8)](https://paperpile.com/c/FZKGcV/aAyp) | celltype |

**Table 9: Dataset information on cell filtering and annotations.** Specifies if the quality controls on the cells of each dataset have been already performed or if it is present in the retrieved object. Column Version QC indicates the version of the dataset where QC is available. Cell Annotation column indicates which datasets have a ground-truth available.

### Reviewer #2: The manuscript ‘Curated Single Cell Multimodal Landmark Datasets for R/Bioconductor’ described an effort to compile landmark datasets from various of single-cell multi-modal technologies in one place. Specifically, the authors created an R/Bioconductor package, SingleCellMultiModal that allows the easy access to these datasets. This is a valuable resource for method developer and for experimentalist who might be thinking about generating the multi-modal datasets.

We thank the reviewer for their positive evaluation of our work.

### Major concern: 1. For method developers who want to test the method using a publicly available dataset, they will need to have the ground-truth annotation associated with the data. For most of the datasets described in the manuscript, a common ground-truth information one would need is the cell type annotation. It’d be clearer if the authors have more explicit description on what metadata is provided for each dataset.

We thank the reviewer for raising this important point and we agree that datasets with manually annotated cell type information are extremely useful for method development. Please see Reviewer #1 Point 1 for our response to this comment.

### 2. For some of the data types, such as 10X multiome and seqFISH datasets, there are additional datasets critical for the analysis. For example, the fragment file (listing the open fragment region sequenced per cell) for 10X multiome, and the fluorescence images for seqFISH. How would these data be distributed to the users?

We thank the reviewer for raising this important point. Unfortunately, the seqFISH fluorescence images are not available in the original paper.

In addition, these files, e.g., the original high-resolution images of seqFISH and the fragment files of 10X Multiome, are mostly used in the preprocessing of these data, whereas we focus on pre-processed data matrices. However, the package documentation for the 10x Multiome dataset links to the 10x Genomics official website with a link to the included dataset, where the fragments files are publicly available.

### 3. Currently, it is difficult to conceptualize how an object from the SingleCellMultiModal package looks like. I think adding a more detailed description and potentially a graphical illustration of the object under the ‘Summary of landmark datasets in SingleCellMultiModal’ heading will be beneficial for readers.

We think it would be easier to conceptualize our work in light of another paper from our group, “Software For The Integration Of Multi-Omics Experiments In Bioconductor.” Ramos M et al (2017) [doi: 10.1158/0008-5472.CAN-17-0344]. MultiAssayExperiment harmonizes data management of multiple experimental assays performed on an overlapping set of specimens. Although originally developed for patient data from multi-omics cancer studies, the MultiAssayExperiment framework naturally applies also to single cells. So, in this context, “patients” in Figure 1 would be replaced by “cells”. It also provides a familiar Bioconductor user experience by extending concepts from SummarizedExperiment, supporting an open-ended mix of standard data classes for individual assays, and allowing subsetting by genomic ranges or rownames. See also <https://www.bioconductor.org/packages/release/bioc/vignettes/MultiAssayExperiment/inst/doc/MultiAssayExperiment.html>.

Figure 1: **MultiAssayExperiment object schematic shows the design of the infrastructure class**

The colData provides data about the patients, cell lines, or other biological units, with one row per unit and one column per variable. The experiments are a list of assay datasets of arbitrary class, with one column per observation. The sampleMap links a single table of patient (or cells in our work) data (colData) to a list of experiments via a simple but powerful table of experiment:patient edges (relationships), that can be created automatically in simple cases or in a spreadsheet if assay-specific sample identifiers are used. sampleMap relates each column (observation) in the assays (experiments) to exactly one row (biological unit) in colData; however, one row of colData may map to zero, one, or more columns per assay, allowing for missing and replicate assays. Green stripes indicate a mapping of one subject to multiple observations across experiments.

### Minor concern: 1. For Figure 1A and 1B, enlarge the font. Currently, I need to zoom in quite a lot to see the text in the figures. For the x-axis labels, could enlarge the font and rotate them to avoid stacking.

Thank you for identifying this readability problem, we have improved Figures 1A and 1B accordingly.

Figure 1A:

Figure 1B:

### 2. There is a typo in the Figure 4 legend. Should be ‘(right) pre-processing steps of source data’.

Thank you for noticing, we also have corrected this error.
